# “Cancer – Educate to Prevent” – High-School Teachers, the New Promoters of Cancer Prevention Education Campaigns

**DOI:** 10.1371/journal.pone.0096672

**Published:** 2014-05-09

**Authors:** Ana Barros, Luís Moreira, Helena Santos, Nuno Ribeiro, Luís Carvalho, Filipe Santos-Silva

**Affiliations:** 1 Public Awareness of Cancer Unit, Cancer Institute of Pathology and Molecular Immunology of University of Porto – Ipatimup, Porto, Portugal; 2 Faculty of Sciences of University of Porto – FCUP, Porto, Portugal; 3 Department of Research Methodology and Data Analysis, Health School of Vila Nova de Gaia – Piaget Institute, Vila Nova de Gaia, Portugal; 4 Autonomous Section of Social Sciences, Faculty of Economics of the University of Porto – FEP, Porto, Portugal; 5 Department of Pathology and Oncology, Faculty of Medicine of the University of Porto – FMUP, Porto, Portugal; University of Tennessee, United States of America

## Abstract

Cancer is one of the leading causes of death worldwide, and thus represents a priority for national public health programs. Prevention has been assumed as the best strategy to reduce cancer burden, however most cancer prevention programs are implemented by healthcare professionals, which constrain range and educational impacts.

We developed an innovative approach for cancer prevention education focused on high-school biology teachers, considered privileged mediators in the socialization processes. A training program, “Cancer, Educate to Prevent” was applied, so that the teachers were able to independently develop and implement prevention campaigns focused on students and school-related communities. The program encompassed different educational modules, ranging from cancer biology to prevention campaigns design. Fifty-four teachers were empowered to develop and implement their own cancer prevention campaigns in a population up to five thousands students.

The success of the training program was assessed through quantitative evaluation – questionnaires focused on teachers' cancer knowledge and perceptions, before the intervention (pre-test) and immediately after (post-test). The projects developed and implemented by teachers were also evaluated regarding the intervention design, educational contents and impact on the students' knowledge about cancer. This study presents and discusses the results concerning the training program “Cancer, Educate to Prevent” and clearly shows a significant increase in teacher's cancer literacy (knowledge and perceptions) and teachers' acquired proficiency to develop and deliver cancer prevention campaigns with direct impact on students' knowledge about cancer.

This pilot study reinforces the potential of high-school teachers and schools as cancer prevention promoters and opens a new perspective for the development and validation of cancer prevention education strategies, based upon focused interventions in restricted targets (students) through non-health professionals (teachers).

## Introduction

Cancer is a major worldwide public health problem being the control of cancer incidence and mortality rates a significant challenge to national health systems [1]–[7]. Cancer prevention is nowadays assumed as the most effective strategy to address this public health problem, with some authors referring cancer as *the most preventable and the most curable of major chronic life-threatening diseases* [6]. Cancer education programs that raise the awareness for risk factors and promote healthy lifestyles among general audiences are fundamental initiatives in primary prevention [8]. Unfortunately, comprehensive studies designed to identify target groups and/or social environments (family, school, workplace) predisposed to priority interventions are uncommon as well as studies addressing evaluation of educational impacts [9].

The school system is a privileged socialization instance. In fact, studies demonstrate that schools have the capability and the necessary tools to provide a positive impact on students' health [10], [11]. Teachers are *active* social mediators [12] and thus they are key players for cognitive and practical (behavioral) changes. They are the main agents of school socialization and they are invested to perform a triangulated mediation, interacting with the school, the students and the families. A previous study conducted in 1989, at primary and secondary schools of 12 European Union countries demonstrated the potential of the teachers in health education at schools, namely on cancer prevention [13]. More than two decades after that study, experimental research evaluating the feasibility of a cancer prevention education model based upon teachers, both in Portugal and all over Europe, remains to be done.

Regardless of the schools potential to promote Cancer Education programs in local communities, so far this task has been assigned to healthcare professionals from institutions, such as universities, public health schools, medical centers and other cancer related organizations. Most of these interventions are local, uncoordinated and without any follow up on educational impact [10], [14], [15].

As it is known, more than half of all cancer deaths can be attributed to wrong behavioral options [16]. Consequently our nuclear argument is that cancer prevention education programs centered on school-based interventions may be more efficiently delivered to larger audiences, and with enhanced impact on long-term behavioral changes. Our hypothesis is that biology teachers can be successfully trained to independently develop and promote relevant cancer prevention education programs in schools. Our research was focused on evaluating the feasibility of training high school biology teachers educational skills on cancer prevention, so they will be able to develop their own materials and implement impactful cancer prevention campaigns in schools. The program “Cancer, Educate to Prevent” is an innovative approach for cancer prevention education, which trains the teachers to: a) learn the basic principles of cancer biology, epidemiology and prevention; b) select, validate and organize relevant information (e.g. scientific literature databases); plan and implement prevention campaigns at schools. The results obtained clearly showed that perceived and real knowledge about the different cancer topics, significantly increase in trained teachers. Additionally, enrolled teachers have been able to produce and deliver impactful cancer prevention campaigns among their school communities with significantly increase in students' knowledge about cancer that reached an estimated public of five thousand people. Given that the trained teachers reflect the general profile of Portuguese Biology teachers, this pilot study reinforces the potential of teachers and schools as cancer prevention promoters and opens a new perspective for a nation-wide strategy on cancer prevention education.

## Methodology

### Training program

During 2012, we carried out a training program “Cancer, Educate to Prevent” for biology teachers, certified by the Portuguese Ministry of Education and Science and promoted by health education specialists from Ipatimup (Institute of Pathology and Molecular Immunology of University of Porto). Sixty-two teachers from schools of the North and Centre of Portugal were voluntary enrolled in this program. Although it is a small sample for theoretical statistical purposes, it is a representative sample for our research goals (indeed, it's the maximum number of participants the program could deal with, considering all the research process and methodological strategies).

The training program was focused on five of the most incident cancers in Portugal: colorectal, gastric, breast, cervical and skin cancer and encompassed 20 hours of e-learning sessions (on Moodle platform) and 5 hours of classroom sessions at Ipatimup. The program was structured in 5 training modules: Module 1: Introduction (classroom session); Module 2: Basics of Cancer Biology (e-learning sessions with video casts); Module 3: Prevention (e-learning sessions); Module 4: Development of cancer prevention projects to be implemented at schools; and Module 5: Final session, insight into strategies for cancer awareness and prevention (classroom session). This program had 25 hours of effective training, plus the production and implementation of the cancer prevention education projects developed by the teachers', which on practice has meant that this initiative had a total duration of 4 months.

During the training program, all the participants were continuously evaluated through individual tests performed at the end of every e-learning session. Finally, in the last session teachers were tested about the basic principles of cancer biology and cancer prevention. The extensive evaluation scheme allowed the trainees to optimize the training process according to their own individual characteristics.

### Instruments for data collection - characterization and assessment

Apart from direct observation all along the program, we collected the data using four questionnaires: 1) “Trainees characterization” (See Questionnaire S1); 2) “Trainees perception and knowledge about cancer” (See Questionnaire S2); 3) “Trainees assessment on the training Program” (See Questionnaire S3); and 4) “Students knowledge about cancer and socio-biographic characterization” (See Questionnaire S4). The first one included 32 items organized in three sections: i) Characteristics of other training programs attended in the last three years (11 items); ii) Information on this specific training program (3 items); and iii) Personal and professional data (18 items). The second one included 34 items also organized in three sections: i) Trainees perceptions on population cancer knowledge (3 items); ii) Trainees self-perceptions on cancer knowledge (11 items); and iii) Trainees knowledge on cancer (20 items). The items about trainees' self-perception and knowledge about cancer were organized in four main themes: Cancer Biology, Cancer Prevention, Cancer Epidemiology and Scientific literature databases. The third questionnaire included 29 items organized in three sections too: i) Program structure and organization assessment (19 items); ii) Program impact assessment (6 items); and iii) Program accomplishments on trainees' expectations assessment (4 items). The fourth questionnaire included 19 items and was organized in two sections: i) Students knowledge on cancer with 16 items and ii) Students socio-biographic characterization that included 3 items.

### Study Design and Data Analysis

This pilot study followed a *quasi-experimental* design, with a pre-test before the intervention and a post-test after its conclusion [17]. At the beginning of the program, in the first classroom session, we applied the questionnaire “Trainees characterization” and the questionnaire “Trainees perception and knowledge about cancer”, in a paper format (pre-test). In the last classroom session, we applied again the second questionnaire (post-test). After the end of the program, the questionnaire “Trainees assessment on the training program” was applied online, at the Moodle platform. The questionnaire “Students knowledge about cancer and socio-biographic characterization” was applied in a paper format, both on experimental and on control groups before the implementation of the prevention campaigns designed by teachers (pre-test) and immediately after the intervention (post-test) (see questionnaires in supporting information).

This pilot study was approved (accredited) by two different review boards of Portuguese Ministry of Education and Science: a) The Scientific and Pedagogical Council for Continuous Education and b) The System for Monitoring Schools Surveys. All the participants (teachers and in the case of the students, their parents or tutors) have provided their written informed consent to participate in this study.

Data from surveys were analyzed using IBM SPSS Statistics, version 21. The distribution analysis of the variables under consideration revealed that these couldn't be considered normally distributed. Thus, we opted for the use of nonparametric tests (*Related Samples Friedman's Two-Way Analysis of Variance by Ranks*, *Related-Samples* Wilcoxon Signed Rank Test and *Independent-Samples* Mann-Whitney U Test).

### Student sampling

A total of 1,648 students spread over 82 classes were directly involved in the projects implemented by the 54 teachers that finished the training program.

We randomly selected - by cluster sampling - 21 of these classes to include in the experimental group (a total of 490 students out of 1,648), according to the following inclusion criteria: classes from public schools attending to the 8th, 10th or 11th grade – in order to ensure a 1 year follow-up (9th and 12th grade students' conclude a study cycle and might move to a different school). Besides, the number of classes selected from each geographic region was defined accordingly to its demographic density.

After defining the experimental group we selected 13 classes (a total of 298 students) to include in the control group. These classes were selected according to the same inclusion criteria defined to the experimental group, from the same regions (specifically from the same districts), with similar social, economic and demographic characteristics in terms of context, which had any kind of participation in this project (any teachers of these schools were involved in the training program).

At the end of the program we had a drop out of 3 classes on the experimental group and 2 classes on the control group, resulting in a sample of 18 classes in the experimental group (385 students) and 11 classes in the control group (236 students).

## Results

### Teachers

#### Sample characterization

The questionnaire of “Trainees characterization” showed that of the 62 biology teachers that participated in the training program, 88.7% (55) are females and 87.1% (54) have less than 50 years old (more information on teachers' personal data, Table S1). Most of them have a stable professional status, since 83.9% (52) have 11 or more years of service and already belong to the school staff. Also, 88.7% (55) of the trainees teach in public schools from North or Center region of Portugal, 74.2% (46) teach between 19 and 22 hours a week and 83.9% (52) perform other activities in school (e.g. management and administration) (more information on teachers' professional data, Table S2). The trainees were also asked about their involvement in other professional activities, specifically in health related jobs and 95.2% (59) answered that they never worked in this area before (more information on teachers' training profile, Table S3).

**Figure 1:**
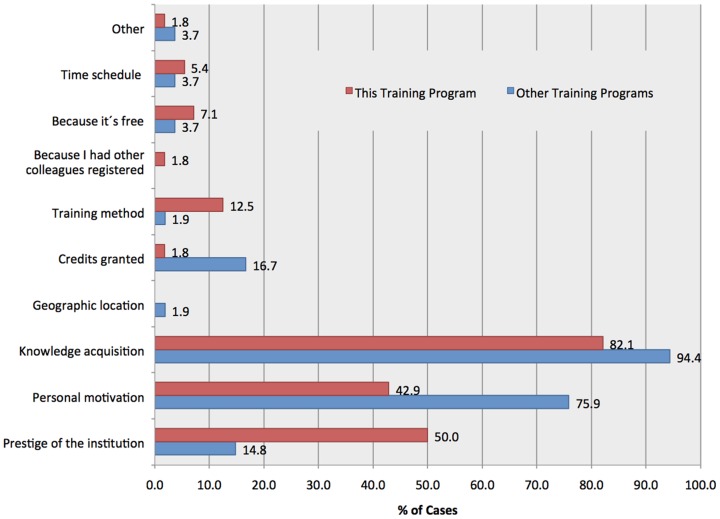
Reasons why teachers choose the training programs attended before the academic year 2011/2012 and the training program Cancer, Educate to Prevent (n = 62). The main reasons selected by teachers to participate in the training programs before 2011/2012 were: 94.4% (51) knowledge acquisition; 75.9% (41) personal motivation (71%) and 16.7% (9) credits granted.

Fifty-six teachers (90.3%) took notice of this training program by e-mail, and the remaining by other colleagues (by word of mouth). When asked about the main reasons why they decided to participate in the program, 82.1% (46) indicated “knowledge acquisition”, 50% (28) mentioned the “prestige of the institution Ipatimup”, and 42.9% (24) indicated “personal motivation”. Knowledge acquisition was also identified by 94.4% (51) of the teachers, as the main reason that motivated them to enroll training programs before 2011/2012 (Figure 1). Personal motivation was pointed out as the second most important reason, by 75.9% (41) of the teachers. Fifty-four (87.1%) out of the 62 teachers that enrolled the training program completed it with success, and 8 (12.9%) of them dropped out during the e-learning sessions and project implementation phase.

#### Perceptions and knowledge about cancer

*Pre-test*: The questionnaire “Trainees perception and knowledge about cancer” applied at the beginning of the training program (*pre-test*), showed that the teachers had a perception level of 56.8% on Cancer Biology, 61.8% on Cancer Prevention, 38.8% on Cancer Epidemiology and 36.4% on Scientific Literature Databases (Table 1).

**Table 1:** Pre-test and post-test results on teachers' self-perception and knowledge about cancer (n = 56).

		Pre-test		Post-test		Post-test - Pre-test	
		Mean (%)	SD	Mean (%)	SD	Dif.^c^ (%)	p-value^a^
Perceptions	Cancer Biology	56.8	18.8	86.3	12.1	29.5	<0.001
	Cancer Prevention	61.8	21.1	92.7	8.8	30.9	<0.001
	Cancer Epidemiology	38.8	21.8	86.3	13.0	47.5	<0.001
	Scientific Literature Databases	36.4	22.8	85.2	13.5	48.8	<0.001
	Global^b^	53.5	17.0	86.8	10.8	33.4	<0.001
Knowledge	Cancer Biology	51.0	18.0	87.7	6.3	36.7	<0.001
	Cancer Prevention	81.7	19.4	98.9	4.5	17.2	<0.001
	Cancer Epidemiology	56.3	23.0	89.3	18.1	33.0	<0.001
	Scientific Literature Databases	43.8	34.5	99.1	6.7	55.4	<0.001
	Global^b^	60.1	12.1	91.9	4.5	31.8	<0.001

a Pre-test *versus* Post-test: Related-Samples Wilcoxon Signed Rank Test.

b Overall weighted mean (according to the number of items in each topic).

c Difference between the Post-test Mean and the Pre-test Mean (in %).

Comparing the levels of perception on the four topics (based on *Friedman's Two-Way Analysis of Variance by Ranks*) we conclude significant statistical differences among them (p<0.001, Table 2). The *Pairwise Analysis* allows us to identify which specific topics significantly differ from each other (Table 2). According to this analysis, the perception levels on Cancer Biology are significantly higher than the levels on Cancer Epidemiology (p<0.001) and Scientific Literature Databases (p<0.001); and the perception levels on Cancer Prevention are significantly greater than the levels on Cancer Epidemiology (p<0.001) and Scientific Literature Databases (p<0.001).

**Table 2:** Topics comparison: Perceptions, Knowledge, Pre-test and post-test (n = 56).

		Pre-test				Post-test			
		p-value^a^	p-values^b^			p-value^a^	p-values^b^		
			CB*	CP*	CE*		CB*	CP*	CE*
Perceptions	Cancer Prevention	<0.001	1.000	–	–	<0.001	0.001	–	
	Cancer Epidemiology		<0.001	<0.001	–		1.000	<0.001	–
	Scientific Literature Databases		<0.001	<0.001	1.000		1.000	<0.001	1.000
Knowledge	Cancer Prevention	<0.001	<0.001	–		<0.001	<0.001	–	
	Cancer Epidemiology		0.265	<0.001	–		<0.001	0.069	–
	Scientific Literature Databases		1.000	<0.001	0.554		<0.001	1.000	0.036

a Related Samples Friedman's Two-Way Analysis of Variance by Ranks.

b Corrected p-values from Pairwise Tests.

*CB – Cancer Biology, CP – Cancer Prevention, CE – Cancer Epidemiology.

The assessment on trainees' knowledge revealed levels of 51.0% on Cancer Biology, 81.7% on Cancer Prevention, 56.3% on Cancer Epidemiology and 43.8% on Scientific Literature Databases.

Comparing the levels of knowledge on the four topics we conclude significant statistical differences among them (p<0.001, Table 2). According to the *Pairwise Analysis* (Table 2), the level of knowledge on Cancer Prevention is significantly higher than the correspondent level on each of the other three topics (all the p-values <0.001). These differences range from 25.4% to 37.9%.

In Table 3 we compare the perception levels to knowledge levels at the beginning of the program. In general, the levels of knowledge are higher than the levels of perception. The topic related to Cancer Biology is the only exception, where perception is above knowledge. Despite this difference is statistically significant (p = 0.043), is only 5.8%. The knowledge level on Cancer Prevention is 19.9% above the perception level and on Cancer Epidemiology this difference is 17.5% (both of these differences are statistically significant, with p-values <0.001). The level of perception on Scientific Literature Databases is 7.3% below the knowledge level and this difference is not statistically significant (p = 0.168). On the overall assessment, despite of statistically significant (p = 0.003), the difference between knowledge and perception levels is only 6.6%.

**Table 3:** Teachers' Perception *versus* Knowledge (n = 56).

	Pre-Test		Post-Test	
	Knowledge % - Perception %	p-value a	Knowledge % - Perception %	p-value a
Cancer Biology	-5.8	0.043	1.4	0.778
Cancer Prevention	19.9	<0.001	6.3	<0.001
Cancer Epidemiology	17.5	<0.001	3.0	0.331
Scientific Literature Databases	7.3	0.168	13.9	<0.001
Global b	6.6	0.003	5.0	0.001

a Related-Samples Wilcoxon Signed Rank Test.

b Overall weighted mean (according to the number of items in each topic).

*Post-test*: The questionnaire “Trainees perception and knowledge about cancer” applied at the end of the training program (*post-test*), showed that the levels of perception were 86.3% on Cancer Biology, 92.7% on Cancer Prevention, 86.3% on Cancer Epidemiology and 85.2% on Scientific Literature Databases (Table 1).

Comparing the levels of perception on the four topics, we conclude significant statistical differences among them (p<0.001, Table 2). According to the *Pairwise Analysis* (Table 2), the perception level on Cancer Prevention is significantly higher than the correspondent level on each of the other three topics (all the p-values ≤ 0.001), although this differences only ranges from 6.4% to 7.5%.

The levels of knowledge were 87.7% on Cancer Biology, 98.9% on Cancer Prevention, 89.3% on Cancer Epidemiology and 99.1% on Scientific Literature Databases (Table 1).

Comparing the levels of knowledge on the four topics we conclude significant statistical differences among them (p<0.001, Table 2). According to the *Pairwise Analysis* (Table 2), the level of knowledge on Cancer Biology is significantly lower than the correspondent level on each of the other three topics (all the p-values <0.001). These differences range from 1.6% to 11.4%. The level of knowledge on Cancer Epidemiology is significantly lower than the correspondent level on Scientific Literature Databases (p = 0.036).

In Table 3 we compare the perception levels to knowledge levels at the end of the program. The levels of knowledge are higher than the levels of perception in all topics. These differences are 1.4% on Cancer Biology and 3.0% on Cancer Epidemiology, both with no statistical significance (p = 0.778 and p = 0.331, respectively). On Cancer Prevention this difference is 6.3% and 13.9% on Scientific Literature Databases, both statistically significant (with p-values <0.001). The global difference between knowledge and perception is statistically significant (p = 0.001), but is only 5.0%.

**Figure 2:**
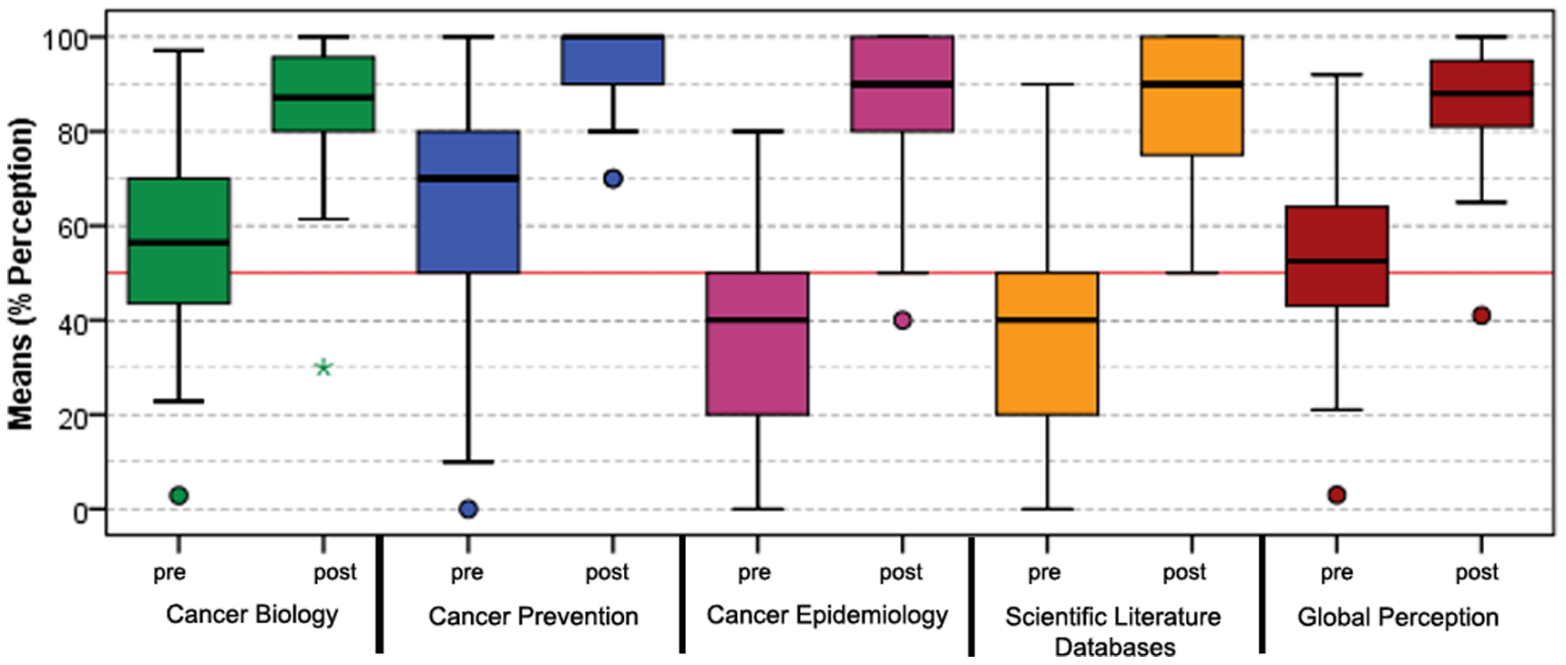
Teachers' self-perceptions about cancer. This figure shows the teachers' self-perceptions regarding the pre-test and the post-test. Results are shown in four main subjects (Cancer Biology, Prevention, Epidemiology and Scientific Literature Databases and Global perception).

**Figure 3:**
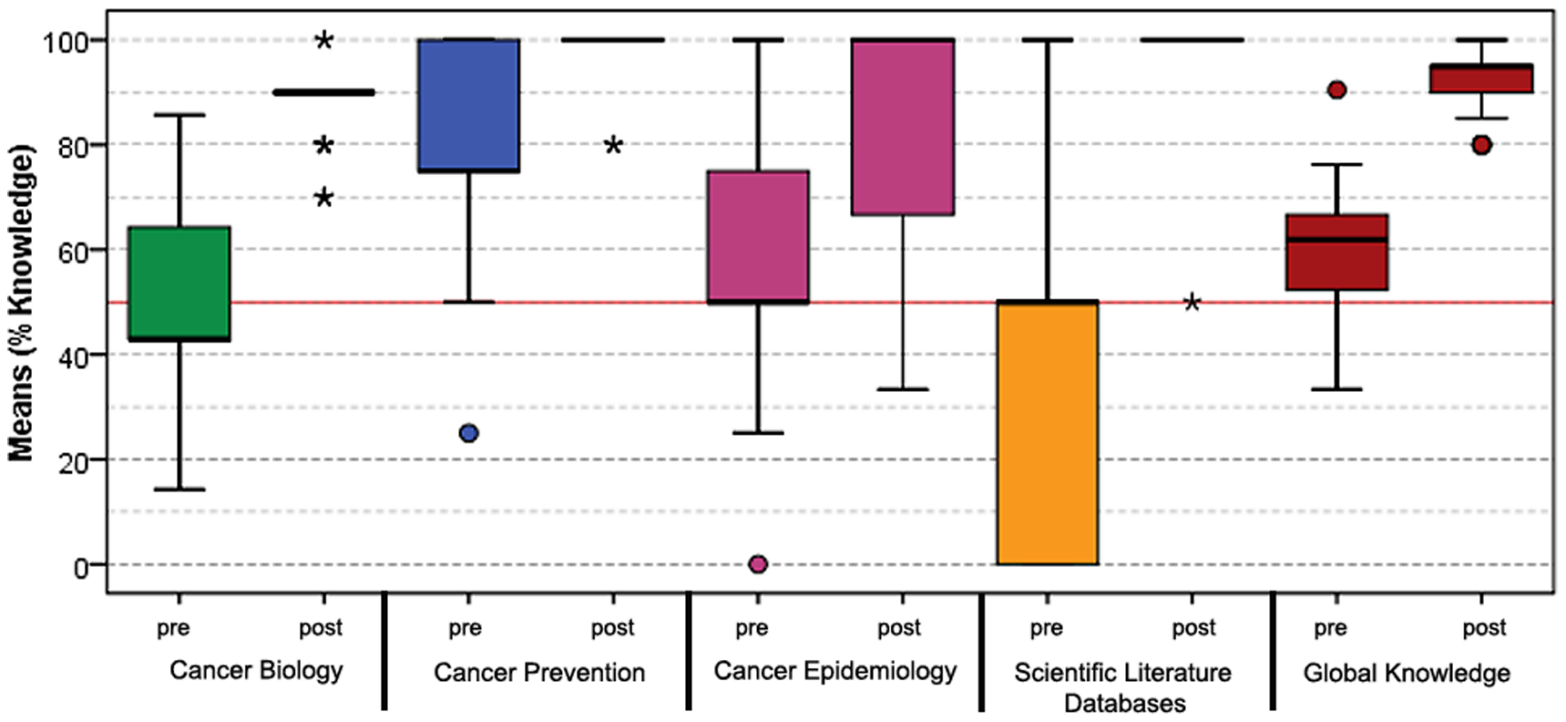
Teachers' knowledge about cancer. This figure shows the teachers' knowledge regarding the pre-test and the post-test. Results are shown in four main subjects (Cancer Biology, Prevention, Epidemiology and Scientific Literature Databases and Global perception).

*Pre-test versus Post-test*: Comparing the post-test with the pre-test results we can conclude a significant increase on the trainees self-perceptions and knowledge at the end of the training program, in each of the four topics and in the overall assessment (all the p-values <0.001, Table 1). Cancer Biology increased 29.5% on self-perceptions and 36.7% on knowledge; Cancer Prevention increased 30.9% on self-perceptions and 17.2% on knowledge; Cancer Epidemiology increased 47.5% on self-perceptions and 33.0% on knowledge; and Scientific Literature Databases increased 48.8% on self-perceptions and 55.4% on knowledge. At last, the overall assessment increased 33.3% on self-perception and 31.8% on knowledge. These results are presented in Table 1, Figure 2 and Figure 3. The dropout rate at this training stage was 9.7% (6 teachers out of 62 that started the program).

### Cancer prevention education projects developed and implemented by teachers

Ninety six percent (54 out of 56) of the teachers that completed the training program have also achieved the implementation of their own cancer prevention education projects at their schools. Cancer prevention projects were focused on breast, cervical, skin and colorectal cancer.

A total of 1,648 students from 82 middle and high school classes, were directly involved in the projects, 72.2% (39) of the teachers implemented their project with high school students and only 27.8% (15) with middle school students.

Almost all the projects implemented, 88.9% (48) requested the active participation of the students, as the primary target of these campaigns. Students were engaged in several events, from seminars to laboratory and outdoor activities, which provided a greater interaction between teachers and students, a critical point for the success of these actions. In the cancer prevention education projects, 77.8% (42) of the teachers used oral presentations/seminars, 79.6% (43) used printed materials (posters or leaflets), 38.9% (21) used audiovisual contents, and 11.1% (6) lab activities. Moreover, 25.9% (14) of the projects had a contribution or intervention of external healthcare professionals (nurses, medical doctors and pharmacists) while 57.4% (31) implemented innovative approaches such as roleplaying activities, outdoor activities and healthy eating demonstrations. Besides involving directly their students, trained teachers' extended the intervention through the entire schools communities reaching an estimated total of five thousand students. It is also important to emphasize that these cancer prevention projects, due to produced materials and activities, exceed the school context, reaching families and local communities (data not shown).

#### Training Program Evaluation

The questionnaire “Trainees assessment on the training program”, applied at the end of the training, was answered by 85.5% (53) of the participants. All the trainees agreed about the coherence of contents presentation while 98.1% (52) agreed about its relevance. In which concerns the adopted methodologies, 92.5% (49) of the trainees agreed that they were appropriate and motivational and all the trainees agreed about the effectiveness of the support provided by the trainers.

In which refers to the adequacy of the training methods, only 7.5% (4) of the individuals considered that the training methodology was not adequate neither stimulating, while 9.4% (5) consider the assessment methods of the training program inadequate. Moreover, 56.6% (30) of the respondents considered the duration of the training program appropriate while 34.0% (18) considered it too short and 9.4% (5) considered it too long.

About the impact of this training program, all the trainees considered it as relevant or very relevant to teachers' personal development and 98.2% (52) considered that it increased their social/civic responsibility. Also 98.2% (52) of the trainees considered it relevant or very relevant to increase knowledge about cancer prevention (both for them and their students). In the answers obtained about behavior changes of the teachers' and their students towards cancer prevention, 88.6% (47) considered the contribution to their own behavior change as relevant or very relevant while, for their students, 92.4% (49) considered it also relevant or very relevant.

Forty-six teachers (87%) claimed that the training program either met or was above their previous expectations with 13% (7) claiming that it was below the expectations. Finally, 96.2% (51) registered that they would recommend this training program to their peers.

### Students

#### Sample characterization

The experimental group has 18 classes from 19 public schools from the North or Center region of Portugal, with a total of 385 students. This group is well balanced by gender, with 54.3% (209) females and 45.7% (176) males. The mean age is 15.2 years old; 26.8% (103) are attending middle school (8^th^ grade), while the high-schoolers are 34.8% (134) of the 10^th^ grade; and 38.4% (148) of the 11^th^ grade.

The control group has 11 classes from 5 public schools, with a total of 236 students. In this group, 54.7% (129) are males and 45.3% (107) are females. The mean age is 15.1 years old; 27.5% (65) are attending middle school (8^th^ grade), while high-schoolers 47.0% (111) of the 10^th^ grade and 25.4% (60) of the 11^th^ grade.

#### Knowledge about cancer

*Pre-test*: The questionnaire “Students knowledge about cancer and socio-biographic characterization” applied before the implementation of the projects (*pre-test*), showed that the cancer knowledge levels in experimental group were 54.1% for Cervical Cancer, 58.3% for Breast Cancer, 32.1% for Colorectal Cancer and 60.3% for Skin Cancer, while for the control group the levels were 40.5% for Cervical Cancer, 52.3%, for Breast Cancer, 20.7% for Colorectal Cancer and 60.6% for Skin Cancer (Table 4). The overall knowledge was 43.6% (Table 4).

**Table 4:** Pre-test and post-test results on Students' knowledge about cancer.

	Experimental Group (N = 385)						Control Group (N = 236)						
	Pre-test		Post-test		Post-test - Pre-test		Pre-test		Post-test		Post-test - Pre-test		
	Mean (%)	SD	Mean (%)	SD	Dif.^c^ (%)	p-value^a^	Mean (%)	SD	Mean (%)	SD	Dif.^c^ (%)	p-value^a^	p-value^d^
Cervical Cancer	54.1	32.0	56.8	32.0	2.7	0.071	40.5	26.0	45.7	28.4	5.2	0.001	0.374
Breast Cancer	58.3	22.2	62.9	21.0	4.6	<0.001	52.3	19.2	55.1	20.5	2.8	0.058	0.343
Colorectal Cancer	32.1	23.5	39.9	28.0	7.8	<0.001	20.7	18.3	22.6	19.3	1.9	0.153	0.012
Skin Cancer	60.3	22.1	66.4	23.6	6.2	<0.001	60.6	23.8	59.9	24.0	-0.7	0.680	0.006
Global b	51.3	15.4	56.7	16.5	5.3	<0.001	43.6	13.7	45.9	15.5	2.3	0.006	0.009

a Intra-group comparison: Pre-test *versus* Post-test (Related-Samples Wilcoxon Signed Rank Test).

b Overall weighted mean (according to the number of items in each topic).

c Difference between the Post-test Mean and the Pre-test Mean (in %).

d Inter-group comparison: Difference in Experimental Group *versus* Difference in Control Group (Independent-Samples Mann-Whitney U Test).

*Post-test*: The questionnaire “Students knowledge about cancer and socio-biographic characterization” applied after the implementation of the projects (*post-test*), showed that the cancer knowledge levels in the experimental group were

56.8% on Cervical Cancer, 62.9% on Breast Cancer, 39.9% on Colorectal Cancer and 66.4% on Skin Cancer. The overall knowledge was 56.7% (Table 4).

On the control group, the levels of knowledge were 45.7% on Cervical Cancer, 55.1% on Breast Cancer, 22.6% on Colorectal Cancer and 59.9% on Skin Cancer. The overall knowledge was 45.9% (Table 4).

*Pre-test versus Post-test*: Comparing the post-test with the pre-test results in the experimental group (*intra-group comparison*), we can conclude a significant increase on cancer knowledge in three of the four topics: 4.6% on Breast Cancer, 7.8% on Colorectal Cancer and 6.2% on Skin Cancer (all the p-values <0.001, Table 4). The knowledge on Cervical Cancer increased 2.7%, but it wasn't statistically significant (p = 0.071, Table 4). The overall knowledge increased 5.3% (p-value <0.001, Table 4). On the control group, we can conclude no significant changes in three of the four topics: 2.8% on Breast Cancer (p-value = 0.058), 1.9% on Colorectal Cancer (p-value = 0.153) and -0.7% on Skin Cancer (p-value = 0.680). The knowledge on Cervical Cancer had a significant increase of 5.2% (p-value = 0.001, Table 4). The overall knowledge increased 2.3% (p-value = 0.006, Table 4).

Comparing the difference between the post-test and the pre-test in the experimental group (*inter-group comparison*), with the analogous difference in the control group, we can conclude no significant differences in the topics related to the Cervical Cancer (p-value = 0.374) and the Breast Cancer (p-value = 0.343). On the topics related to the Colorectal Cancer and the Skin Cancer, the knowledge increase in the experimental group is significantly higher than in the control group (p-value = 0.012 and p-value = 0.006, respectively, Table 4). The overall knowledge also increased significantly higher in the experimental group than in the control group (p-value = 0.009, Table 4).

## Discussion

In this pilot study we designed and implemented a training program - “Cancer, educate to prevent” - for high-school teachers and we further evaluated the program impact on the trainees cancer-related knowledge and proficiency to develop impactful prevention campaigns. We worked with Biology teachers because: i) as experts in biology, it is expected they will be more *intrinsically* motivated for cancer prevention than other teachers [18]; ii) some of the contents they teach are related to prevention; iii) most of the times, they are responsible for health education programs at schools; iv) they are often the first person that students contact when they have doubts, fears or worries about health, and thus they actively influence students health behaviors [19].

The sixty-two high school Biology teachers that participated in this pilot study constitute a homogeneous group in which concerns socio-demographic (e.g. gender and age) and career characteristics (e.g. years of service, job situation) (Tables S1 and S2). Teachers are mostly females, younger than 50 years old, teaching in middle and high schools, with a stable job situation, which gives them the opportunity to manage long-term projects (Table S2). Overall the teacher's characteristics reflect the profile of “Biology Teachers” population published by the Portuguese Ministry of Education and Science, [20]. It is also clear that the participants share the same motivation profile, given the reasons invoked for participation in this program and the training activities of the last three years (Figure 1). In fact, these individuals actively seek to keep updated with regard to their teaching practice and their commitments as educational agents, which is perceived by the number of previous courses (training programs) attended. The accreditation of training activities attended serves also as an indicator that these teachers look for initiatives relevant for their careers progression. Interestingly, despite the teachers' motivation to attend training activities, only one third of them (21) participated in health-related education trainings (Table S3) with only 3.2% (2) being engaged in extra-curricular health-related activities or jobs. These results reflect the reduced offer of training programs in health education namely in cancer prevention education. Additionally, the existing training programs are promoted by private associations and patients groups being mostly delivered by health professionals. These programs do not have a formal accreditation and thus remain out of teacher's training scope [21]-[23].

At the beginning of the training program the pre-test showed that the teachers already had a basic knowledge about cancer. It is also important to notice that the levels for perception and knowledge are always higher for general topics like Cancer Biology and Cancer Prevention than for more restrict ones like Cancer Epidemiology or Scientific Literature Databases (Table 1, Figures 2 and 3). The level of knowledge is always higher than the level of perception (though not always statistically significant) except for the topic Cancer Biology with perception being higher than knowledge. This result might be explained by the fact that Cancer Biology is included in high schools Biology curriculum [24]. Teachers could be more confident, because they have to teach these contents to their students and they had an academic background in this area. For the topic Cancer Prevention, most of the guidelines are common sense so teachers tend to know about them. The same does not happen for Cancer Epidemiology and Scientific Literature Databases, being the teachers less confident and with lower knowledge for these topics. Assessment of the training impact (post-test) showed that perception and knowledge significantly increased for all the topics, which proves the effectiveness of the methodology. The trainee's perception levels remain below knowledge levels, which might suggest a defensive attitude about the new acquired competences, nonetheless the majority of trainees (96%) were able to conceive and implement cancer prevention campaigns in their schools. Interestingly, some projects involved the entire school, families and local community, which reveal a strong perception of the importance of the social, cultural, economic and environmental contexts for these types of initiatives [25].

The impact of teachers' prevention projects on students' cancer literacy was assessed in a population of 385 students (experimental group), by comparison with a control population of 236 students. The increase of cancer global knowledge was significantly higher in experimental group vs. controls (p = 0.009) (Table 4, inter-group comparison). A detailed analysis of the experimental group (intra-group comparison) showed that students involved in teacher's prevention projects revealed a statistically significant increase in knowledge for Breast, Colorectal and Skin cancers, while there is no significant increase for the Cervical cancer knowledge which might reflect an existing baseline literacy. Cervical cancer and Human Papilloma Virus (HPV) have been, since 2008, the focus of sounding media campaigns promoting HPV vaccination [26], [27], also the Portuguese schools have mandatory Sexual Education programs started before this intervention [28]. Regarding the results obtained for Breast Cancer in the experimental group there is a significant increase in knowledge still lower than that for Colorectal and Skin Cancer, this might be explain by the fact that Breast Cancer is one of the cancers with higher visibility in media education campaigns [29]. The same reasons stated above [26]–[29], can also explain the results obtained in the control group (intra-group comparison). A detailed analysis of the Inter-group comparison showed that, there is a significant increase of Colorectal and Skin cancers knowledge in experimental population vs. controls, while there is no significant difference for Cervical and Breast cancers which might reflect the exposure of students (both experimental and controls) to available existing information on media. To better understand the reasons behind these results it is necessary to expand the study including a characterization of students as health information consumers.

The unique design of this training program, combining theoretical and practical components where teachers have to implement their own projects on the field, clearly contrast with programs from other Portuguese institutions mentioned before [22], [30]. The successful implementation of the prevention campaigns at schools is a relevant indicator about the feasibility of this innovative model of cancer prevention education. It also proves that, with the same basic training program, teachers are capable of independently produce different cancer prevention campaigns with a wide diversity of contents and formats even in demanding conditions (projects were implemented as an extra-curriculum activity, since in Portuguese schools health education is not formal). Furthermore, the impact of the cancer prevention projects promoted by the teachers in schools is undisputable, proving that teachers were capable to transduce the acquire competencies into impactful campaigns with direct effect in students cancer knowledge. Overall, the training program evaluation showed that teachers consider the training very relevant, with the expectations being exceeded, and they would recommend it to colleagues. Comments and suggestions of the trainees summarized in the SWOT Table (Table 5) suggest that podcasts, required work, timing and duration of the training should be optimized in future editions.

**Table 5:** SWOT Table.

Internal origin (Attributes of the system)	Strengths	Weaknesses
	B-learning training.	Timing (period in which the training took place).
	Development of autonomous (and adapted to a specific school community and context) projects to implement at their schools.	Being an extra activity of the school curricula despite the existence of mandatory Health Education programs at Portuguese schools.
	Fast and effective support of the trainers.	Extension and technical language of the podcasts used in e-learning sessions.
	Target population (Biology Teachers).	Amount of work required.
		Short period of time for project implementation in schools.

External origin (Attributes of the environment)	Opportunities	Threats
	Development of a communication network for/between trainees and trainers.	Current socio-economic constrains.
	More editions of this training program to teachers that didn't had the opportunity to participate, with the possibility of participation of teachers from different backgrounds.	Dissatisfaction of teachers towards the teaching career.
	Upgrade this training program for the participants.	Funding of training programs.
	Sharing of the materials/strategies developed by the trainees.	

This table was built considering the evaluation of the training program made by the teachers in which concerns to the strengths and weaknesses, opportunities and threats. It describes some aspects that could be improved in further editions (see Weaknesses) and new ideas that can help teachers to reinforce their role in health education (see opportunities). It is also important to maintain the main structure adopted (methodology) for new editions (see Strengths). The threats found are due to a context of a social and economic crisis that is affecting Portugal.

In conclusion the current research, as a proof-of-concept of an alternative model, showed that high school teachers could be trained to efficiently deliver impactful cancer prevention education campaigns. Considering the obtained results, further lines of research should be explored and extended, namely: a) evaluate the long-term impact of the prevention campaigns delivered by teachers in students cancer literacy and behaviors (ongoing follow-up research); b) evaluate the impact of prevention campaigns delivered by teachers in cancer literacy and behaviors of students' families and local communities; c) evaluate if the training model is transposable to teachers with other academic backgrounds (e.g. arts); d) evaluate if the training model is effective for other diseases (e.g. obesity, diabetes); e) evaluate if the model is nationwide scalable.

## Supporting Information

Questionnaire S1 “Trainees characterization”.
(DOCX)

Questionnaire S2 “Trainees perception and knowledge about cancer”.
(DOCX)

Questionnaire S3 “Trainees assessment on the training program”.
(DOCX)

Questionnaire S4 “Students knowledge about cancer and socio-biographic characterization”.
(DOCX)

Table S1 Socio-demographic characteristics of the teachers' sample.
(DOCX)

Table S2 Teachers career.
(DOCX)

Table S3 Characteristics of the Training Programs attended by teachers in the last three academic years, before 2011/2012.
(DOCX)
