# Supplementary material for: “Cancer – Educate to Prevent” – High-School Teachers, the New Promoters of Cancer Prevention Education Campaigns
Source: PLoS One. 2014 May 9;9(5):e96672. doi: 10.1371/journal.pone.0096672 (PMC4016009; doi:10.1371/journal.pone.0096672)
Supplement: Questionnaire S1 — “Trainees characterization”. (DOCX) [file pone.0096672.s001.docx]

**Questionnaire S1. “Trainees characterization”**

**(32 items organized in three sections)**

| **Question** | | | **Answer options** |
| --- | --- | --- | --- |
| **Section 1 - Characteristics of other training programs attended in the last three academic years before 2011/2012**  **(11 items)** | | | |
| 1. | As a teacher, how many training programs have you attended in the last three academic years? | | - None; - 1; - 2; - 3; - More than 3; - I don´t know how many trainings I attended in the last three academic years. |
| 2. | How many credits have you assured? | | - Less than 1; - Between 1 and 1.99; - Between 2 and 2.99; - Between 3 and 3.99; - Between 4 and 4.99; - 5 or more. |
| 3. | How much money did you spend with training programs attended? | | - All free; - < 100 €; - 100 – 200€; - 200 – 300€; - 200 – 300€; - >300 €. |
| 4. | How many training programs have you attended over 30km from your residence area? | | - None; - 1; - 2; - 3; - More than 3. |
| 5. | Which were the main reasons why you chosen the training programs attended before 2011/2012? (Select only two options) | | - Prestige of the institution; - Personal motivation; - Knowledge acquisition; - Geographic location; - Credits granted; - Training method; - Because I had other colleagues registered; - Because it´s free; - Time schedule; - Other. |
| 6. | What is the nature of the training programs that you attended? | | - Mandatory; - Non-mandatory; - Some are mandatory and others are non-mandatory. |
| 7. | What were the main subjects of the trainings programs that you have attended? (You can select more than one option) | | - Life and Physical Sciences (Biology/Geology); - Educational Sciences; - Teaching practice and Didactics; - Personal Education and Ethics; - Specific training. |
| 8. | What are the institutions that promoted the training programs that you participated? (You can select more than one option) | | - Universities and associated labs; - Public institutions related to the Ministry of Education; - Other public institutions not related to the Ministry of Education; - Other institutions (Non-public). |
| 9. | Did you attend any Health Education training program? | | - Yes; - No. |
| 9.1. | If you attended, what were the main themes (subjects) focused on Health Education Training Programs? (You can select more than one option) | | - Nutrition and Physical activity; - Mental Health and Bullying; - Drugs consumption among teenagers; - Flu A (H1N1); - STD’s (Sexually Transmitted Diseases); - Sexual Education; - Rare Diseases; - Oncological Diseases; - Child Safety; - Other. |
| 10. | Globally, how do you classify the training programs attended before school year 2011/2012 in which concerns to:   - Quality of the contents; - Trainer competence; - Relevance of the knowledge acquired to teaching practice; - Organization and Functioning; - Personal development. | | - Terrible; - Very Bad; - Bad; - Good; - Very good; - Excellent; - No opinion/Don’t know. |
| **Section 2 - Information on this specific training program “Cancer, Educate to Prevent” (3 items)** | | | |
| 11. | | How did you know about this training program? | - Directly, through e-mail; - Indirectly, through e-mail forward; - Indirectly, through other means (poster, conversation with a colleague or a friend). |
| 12. | | Please choose the two main reasons that led you to participate on this training program (Select only two options): | - Prestige of the institution (IPATIMUP); - Personal motivation (I dealt with someone close who has been diagnosed with cancer or I was diagnosed with cancer); - Knowledge acquisition; - Geographic location; - Credits granted; - Training method (e-learning); - Because I had other colleagues registered; - Because it´s free; - Time schedule; - Other. |
| 13. | | What are your expectations toward the training program “Cancer, Educate to Prevent” for:   - Personal development; - Increase of your social responsibility; - Increase the level of your cancer prevention knowledge; - Your personal behavior change towards cancer prevention; - Increase the level of the students’ cancer knowledge; - Students´ behavior change towards cancer prevention. | - Very low; - Low; - High; - Very High. - No opinion/Don’t know. |
| **Section 3 - Personal and professional data (18 items)** | | | |
| 14. | | Gender | - Male; - Female. |
| 15. | | Age | Open-ended question. |
| 16. | | Marital Status | - Single; - Married/Civil Partnership; Divorced/Separated; - Widower. |
| 17. | | Zip Code of your address | Open-ended question. |
| 18. | | Education (highest qualification obtained) | - Bachelor; - Master; - Doctoral. |
| 19. | | If you have an MsC or a PhD degree, please specify the name of the degree and of the institution. | |
| 19.1. | | Master:  Name of the Institution: | Open-ended question. |
| 19.2. | | PhD:  Name of the Institution: | Open-ended question. |
| 20. | | How many schools do you work in this academic year? | - 1; - 2; - 3. |
| 21. | | Please indicate the zip code of your school(s) address and the number of hours per week that you work in each school. | Open ended-question. |
| 22. | | What is your current employment status? | - Term-contract; - School staff. |
| 23. | | How many years of service do you have (until 31 august of 2011)? | Open-ended question. |
| 24. | | Do you perform other activities besides teaching at your school? | - Yes; - No. |
| 24.1. | | If your answer is Yes, what type of activities do you perform?  (You can select more than one option) | - Administration/School board /School management; - Pedagogical activities (position); - Projects management. |
| 25. | | Please indicate the education level where do you teach.  (You can select more than one option) | - Middle school classes; - High school classes; - Vocational educational classes. |
| 26. | | Please indicate the number of classes per grade where do you teach: | Open-ended question. |
| 27. | | Besides teaching, have you ever practiced some professional activity in health area? | - Yes; - No. |
| 27.1. | | If your answer is Yes, Do you still perform this activity with the teaching activity? | - Yes; - No. |
| 28 | | If you want, you can leave your comment using the blank space available on this page. | Open-ended question. |
