# Supplementary material for: “Cancer – Educate to Prevent” – High-School Teachers, the New Promoters of Cancer Prevention Education Campaigns
Source: PLoS One. 2014 May 9;9(5):e96672. doi: 10.1371/journal.pone.0096672 (PMC4016009; doi:10.1371/journal.pone.0096672)
Supplement: Questionnaire S2 — “Trainees perception and knowledge about cancer”. (DOCX) [file pone.0096672.s002.docx]

**Questionnaire S2. “Trainees perception and knowledge about cancer”**

**(34 items organized in three sections)**

| **Question** | | **Answer options** |
| --- | --- | --- |
| **Section 1 – Trainees perceptions on population cancer knowledge (3 items)** | | |
| 1. |  | |
| 1.1. | Population in general | Scale:   - No opinion/Don’t know (0) - Terrible (1); - Very Bad (2); - Bad (3); - Good (4); - Very good (5); - Excellent (6); |
| 1.2. | Peers |  |
| 1.3. | Students |  |
| **Section 2 - Trainees self-perceptions on cancer knowledge (11 items)** | | |
| 2. | For you what is the meaning of the word cancer? | Open-ended question. |
| 3. |  | |
| 3.1. | Oncogene | Scale 1(“ Totally unknown”) -10 (“Totally know”) |
| 3.2. | Tumor Suppressor Gene |  |
| 3.3. | Cell Proliferation |  |
| 3.4. | Angiogenesis |  |
| 3.5. | Apoptosis |  |
| 3.6. | Cell-cell and Cell-matrix adhesion |  |
| 3.7. | Invasion and Metastization |  |
| 3.8. | Cancer Prevention |  |
| 3.9. | Scientific Literature Databases |  |
| 3.10. | Cancer Epidemiology |  |
| **Section 3 - Trainees knowledge on cancer (20 items)** | | |
| 4. | Which of the following expressions is a synonym for the word tumor? | - Hypertrophy; - Hyperplasia; - Tumor; - Metaplasia. |
| 5. | Which of the following options is a possible cause for cancer: | - Viral and bacterial infections; - Excessive consumption of coffee; - Sharing infected needles; - Diet rich in leguminous/ vegetables. |
| 6. | Select the right option: | - The most frequent cancers have hereditary origin; - Cancer is a genetic disease; - Cancer does not occur in tissues with reduced regenerative rates; - Cancer occurs only in tissues with high regenerative rates. |
| 7. | Any gene that encodes a protein whose gain function increases the risk of transformation of a normal cell into a  neoplastic cell is called: | - Proto-oncogene; - Pseudo-gene; - Oncogene; - Tumor Suppressor Gene. |
| 8. | Any gene that encodes a protein whose lost it function increases the risk of transformation of a normal cell into a  neoplastic cell is called: | - Proto-oncogene; - Pseudo-gene; - Oncogene; - Tumor Suppressor Gene. |
| 9. | Which of the following environmental factors is associated to cancer? | - Infrared radiation; - UV radiation; - Sunscreen lotion; - Use of antiperspirants. |
| 10. | Angiogenesis is… | - A process of growth of pre-existing blood vessels; - The development of new blood vessels from pre-existing blood vessels; - The process of substitution of pre-existing blood vessels by new ones; - The development of new blood vessels from pre-existing lymphatic vessels. |
| 11. | Identify the difference between apoptosis and necrosis: | - Necrosis is a reversible process whereas the apoptosis is irreversible; - Necrosis is a process that occurs independently in each cell, whereas on apoptosis it occurs in groups of surrounding cells; - Apoptosis is a process that occurs independently in each cell, whereas on necrosis it occurs in groups of surrounding cells; - Necrosis is an irreversible process whereas the apoptosis is reversible. |
| 12. | The process by which tumor cells permeate other tissues is called: | - Invasion; - Intravasion; - Metastization; - Proliferation. |
| 13. | Which of the following sentences corresponds to the definition of a risk factor? | - Factors that decrease the probability of a person to develop cancer; - Factors that increase the probability of a person to die from cancer; - Factors that increase the probability of a person to develop cancer; - Factors that decrease the probability of a person to die from cancer. |
| 14. | Every measures with the objective to decrease the probability of develop a cancer are called: | - Primary treatment; - Secondary prevention; - Primary prevention; - Secondary prevention. |
| 15. | Which is the section of a scientific article where we can find simultaneously the following information: resume of the work, main methodologies used and main results obtained? | - Introduction; - Conclusion; - Abstract; - Results. |
| 16. | “Globocan” is a scientific literature database of… | - Bibliographic data; - Pharmacological data; - Diseases and clinical cases; - Epidemiological data. |
| 17. | The annual death rate from cancer in a country is the number of ... | - Deaths per year; - New cases per year; - Deaths per year divided by the total population of the country; - New cases per year divided by the total population of the country. |
| 18. | The annual prevalence of cancer in a population refers to the total number of ... | - Cases diagnosed until the moment in that population; - Diagnosed patients who died after a year; - Cases diagnosed multiplied by the the total number of deaths that occurred after one year; - Diagnosed persons who are alive after one year. |
| 19. | Please select from the following myths about cancer which one have support from scientific data: | - Contagious; - Exclusive of the human being; - It is a recent disease (XX century); - The number of cases (frequency) is increasing. |
| 20. | Secondary prevention corresponds to the set of measures that have as objective: | - Cure cancer; - Avoid metastization; - Avoid the cancer; - Early detection of cancer. |
| 21. | Select the incorrect option: | - The metastastization process involves cancer cells that are carried in blood or lymph vessels; - A metastasis can be defined as a neoplastic dissemination; - The metastasis only occurs in the organs of the circulatory and digestive systems; - In the process of metastasis neoplastic cells invade other tissues. |
| 22. | What is the difference between a benign tumor and malignant neoplasm? | - A benign tumor is associated with minor cancers, while malignant tumors are associated with more severe cancers; - A malignant neoplasm is incurable while a benign tumor is curable; - A malignant tumor can invade other tissues whereas benign tumors can not invade; - A malignant neoplasm is unable to detect clinically unlike a benign tumor. |
| 23. | Identify a typical characteristic of cancer cells: | - Sensitivity to growth inhibitory signals; - Capacity to induce angiogenesis; - Incapacity to proliferate; - Sensitivity to apoptosis. |
