# Supplementary material for: “Cancer – Educate to Prevent” – High-School Teachers, the New Promoters of Cancer Prevention Education Campaigns
Source: PLoS One. 2014 May 9;9(5):e96672. doi: 10.1371/journal.pone.0096672 (PMC4016009; doi:10.1371/journal.pone.0096672)
Supplement: Questionnaire S3 — “Trainees assessment on the training program”. (DOCX) [file pone.0096672.s003.docx]

**Questionnaire S3. “Trainees assessment on the training program”**

**(29 items organized in three sections)**

| **Question** | | **Answer options** |
| --- | --- | --- |
| **Section 1 – Program structure and organization assessment (19 items)** | | |
| 1. | The objectives of the training program have been achieved. | - Strongly Disagree; - Moderately Disagree; - Disagree; - Agree; - Moderately Agree; - Strongly Agree. |
| 2. | The contents approached in the training program were relevant. |  |
| 3. | The contents were presented in a coherent and structured way. |  |
| 4. | The training methods used were appropriate. |  |
| 5. | The methodologies used built a way of motivation for the trainees. |  |
| 6. | The support provided by the trainers during the training program was effective. |  |
| 7. | The assessment methods were appropriate and fair. |  |
| 8. | The amount of work required was appropriated. |  |
| 9. | In terms of time, the duration of the training program it was… | - Too short; - Adequate; - Too long. |
| 10. | The number of … sessions was… | |
| 10.1. | online sessions | - Few; - Appropriate; - Too much. |
| 10.2. | classroom sessions |  |
| 11. |  | |
| 11.1. | The theoretical component was… | - Insufficient; - Adequate; - Excessive. |
| 11.2. | The practical component was… |  |
| 12. | The use of the Moodle platform was… | - Extremely Bad; - Very Bad; - Bad; - Good; - Very good; - Excellent. |
| 13. | Overall assessment of: | |
| 13.1. | e-learning sessions (…) | |
| 13.1.1. | Podcasts. | - Extremely Bad; - Very Bad; - Bad; - Good; - Very good; - Excellent. |
| 13.1.2. | Prezi presentations. |  |
| 13.2. | Classroom sessions. |  |
| 14. | Globally, you positively evaluate the… | |
| 14.1. | Performance of trainers in classroom sessions. | - Strongly Disagree; - Moderately Disagree; - Disagree; - Agree; - Moderately Agree; - Strongly Agree. |
| 14.2. | Performance of the speakers in podcasts. |  |
| **Section 2 - Program impact assessment (6 items)** | | |
| 15.1. | Personal development. | - Irrelevant; - Somewhat Relevant; - Relevant; - Very Relevant. |
| 15.2. | Increase of your social responsibility. |  |
| 15.3. | Increase the level of your cancer prevention knowledge. |  |
| 15.4. | Your personal behavior change towards cancer prevention. |  |
| 15.5. | Increase the level of the students’ cancer knowledge. |  |
| 15.6. | Students´ behavior change towards cancer prevention. |  |
| **Section 3 - Program accomplishments on trainees’ expectations assessment (4 items)** | | |
| 16. | Would you recommend this training program to a colleague? | - Yes; - No. |
| 17. | Regarding your expectations on this this training program… | - was below your expectations; - met your expectations; - was above your expectations. |
| 18. | Did you give up on this training program before ending? | - Yes; - No. |
| 18.1. | If your answer is yes to the previous question, please explain briefly the reasons? | Open-ended question. |
