# Supplementary material for: “Cancer – Educate to Prevent” – High-School Teachers, the New Promoters of Cancer Prevention Education Campaigns
Source: PLoS One. 2014 May 9;9(5):e96672. doi: 10.1371/journal.pone.0096672 (PMC4016009; doi:10.1371/journal.pone.0096672)
Supplement: Questionnaire S4 — “Students knowledge about cancer and socio-biographic characterization”. (DOCX) [file pone.0096672.s004.docx]

**Questionnaire S4. “Students knowledge about cancer and socio-biographic characterization”**

**(19 items organized in two sections)**

| **Question** | | **Answer options** |
| --- | --- | --- |
| **Section 1 - Students knowledge about cancer (16 items)** | | |
| 1. | What is the agent responsible for causes cervical cancer? | - *Helicobacter pylori*; - Pseudomonas; - HPV; - HIV; - I don´t know. |
| 2. | Which of the following behaviors increases the risk of infection by HPV? | - Always use condom; - Have several sexual partners; - Sharing needles with infected people; - Using oral contraceptives; - I don´t know. |
| 3. | Taking a vaccine can prevent what type of the following cancers? | - Breast cancer; - Cervical cancer; - Colorectal cancer; - Skin cancer; - I don´t know. |
| 4. | The Pap smear test detects: | - Alterations in cervical cells; - Colon polyps; - Atypical moles; - Breast lumps: - I don´t know. |
| 5. | What is the main cause of the majority of skin cancer cases? | - Hereditary mutations; - Excessive and/or inadequate sun exposure; - Production of vitamin D in excess; - Extended exposure to radioactivity; - I don´t know. |
| 6. | Breast Cancer is a disease that affects ... | - Only men; - Only women; - Mainly men; - Mainly women; - I don´t know. |
| 7. | Which of the following is a breast cancer screening test? | - Biopsy; - Mammography; - Endoscopy; - Cytology; - I don´t know. |
| 8. | What time of day is the most dangerous for sun exposure? | - 7h – 10h; - 16h – 18h; - 9h – 11h; - 11h – 17h; - I don´t know. |
| 9. | Which of these groups have a major risk of developing skin cancer? | - People with fair skin; - People with dark skin; - People with many moles; - All of the options above; - I don´t know. |
| 10. | Which of the following cancer types is the most deadly for women worldwide? | - Breast cancer; - Cervical Cancer; - Colorectal Cancer; - Skin Cancer; - I don’t know. |
| 11. | Which of the following measures decreases the risk of breast cancer for a woman? | - Decrease alcohol consumption; - Decrease the number of sexual partners; - Increase calories intake; - Decrease the use of antiperspirants; - I don´t know. |
| 12. | Which of the following is a colorectal cancer screening test? | - Biopsy; - Mammography; - Colonoscopy; - Cytology; - I don´t know. |
| 13. | There is an increase of the probability to develop colorectal cancer… | - If you have a low fat and red meat diet; - If you has other cases in your family; - If you are infected by *Helicobacter pylori*; - If you are a female; - I don’t know. |
| 14. | Which of the following measures decreases the risk of colorectal cancer? | - Exercise regularly; - Increase calories intake; - Eradication of *Helicobacter pylori*; - Decrease salt consumption; - I don’t know. |
| 15. | What is the most deadly cancer in Portugal? | - Breast Cancer; - Skin Cancer; - Colorectal Cancer; - Cervical Cancer; - I don’t know. |
| 16. | What is the most deadly type of skin cancer? | - Basal cell skin cancer; - Squamous cell skin cancer; - Melanoma; - Sarcoma; - I don’t know. |
| **Section 2 – Students socio-biographic characterization (3 items)** | | |
| 17. | Gender | - Male; - Female. |
| 18. | Age | Open-ended question. |
| 19. | Grade | - 8^th^; - 10^th^; - 11^th^. |
