# Supplementary material for: “Cancer – Educate to Prevent” – High-School Teachers, the New Promoters of Cancer Prevention Education Campaigns
Source: PLoS One. 2014 May 9;9(5):e96672. doi: 10.1371/journal.pone.0096672 (PMC4016009; doi:10.1371/journal.pone.0096672)
Supplement: Table S1 — Socio-demographic characteristics of the teachers' sample. (DOCX) [file pone.0096672.s005.docx]

**Table S1. Socio-demographic characteristics of the teachers’ sample (n=62).**

| **Category** | **Subcategory** | **Result (%)** |
| --- | --- | --- |
| Gender | Male | 7 (11.3) |
|  | Female | 55 (88.7) |
| Age | <30 y | 4 (6.5) |
|  | 30-39 y | 16 (25.8) |
|  | 40-49 y | 34 (54,8) |
|  | ≥50 y | 8 (12.9) |
| Education (highest qualification obtained) | Bachelor | 50 (80.6) |
|  | Master/Doctoral | 12 (19.4) |

The results shown in this table refers to the main socio-demographic characteristics: gender, age and education of the 62 teachers that were selected to participate in the training program “Cancer, Educate to Prevent”.
