# Supplementary material for: “Cancer – Educate to Prevent” – High-School Teachers, the New Promoters of Cancer Prevention Education Campaigns
Source: PLoS One. 2014 May 9;9(5):e96672. doi: 10.1371/journal.pone.0096672 (PMC4016009; doi:10.1371/journal.pone.0096672)
Supplement: Table S2 — Teachers career. (DOCX) [file pone.0096672.s006.docx]

**Table S2. Teachers career (n=62).**

| **Category** | **Subcategory** | **Result (%)** |
| --- | --- | --- |
| Years of service | 1-10 y | 10 (16.1) |
|  | 11-19 y | 28 (45.2) |
|  | ≥20 y | 24 (38.7) |
| Employment status/Working status | Term-contract | 10 (16.1) |
|  | School staff | 52 (83.9) |
| Number of schools where teachers work | 1 | 57 (91.9) |
|  | 2 | 4 (6.5) |
|  | Missing data | 1 (1.6) |
| Sector of schools (number of teachers that work in a…) | Public school | 55 (88.7) |
|  | Private | 3 (4.8) |
|  | Public and Private | 1 (1.6) |
|  | Missing data | 3 (4.8) |
| School-time (Hours/Week) | ≤13 h | 4 (6.5) |
|  | 14-19 h | 7 (11.3) |
|  | ≥20 h | 46 (74.2) |
|  | Missing data | 5 (8.1) |
| Performs other activities besides teaching at school | Yes | 52 (83.9) |
|  | No | 10 (16.1) |

Main characteristics of career of the 62 teachers that were selected to participate in the training program, regarding the academic year 2011/2012.
