# Supplementary material for: “Cancer – Educate to Prevent” – High-School Teachers, the New Promoters of Cancer Prevention Education Campaigns
Source: PLoS One. 2014 May 9;9(5):e96672. doi: 10.1371/journal.pone.0096672 (PMC4016009; doi:10.1371/journal.pone.0096672)
Supplement: Table S3 — Characteristics of the Training Programs attended by teachers in the last three academic years, before 2011/2012. (DOCX) [file pone.0096672.s007.docx]

**Table S3. Characteristics of the Training Programs attended by teachers in the last three academic years, before 2011/2012 (n=62).**

| **Category** | **Subcategory** | **Result (%)** |
| --- | --- | --- |
| Number of training programs attended | None | 3 (4.8) |
|  | 1 | 3 (4.8) |
|  | 2 | 8 (12.9) |
|  | 3 | 17 (27.4) |
|  | >3 | 31 (50) |
| Number of credits granted | <1 | 1 (1.6) |
|  | 1 - 1,99 | 3 (4.8) |
|  | 2 - 2,99 | 15 (24.2) |
|  | 3 - 3,99 | 20 (32.3) |
|  | 4 - 4,99 | 7 (11.3) |
|  | ≥ 5 | 13 (21) |
|  | Not attended any training program | 3 (4.8) |
| Expenses with training programs | All free | 18 (29) |
|  | < 100 € | 9 (14.5) |
|  | 100 – 200€ | 20 (32.3) |
|  | 200 – 300€ | 4 (6.5) |
|  | > 300€ | 8 (12.9) |
|  | Not attended any training program | 3 (4.8) |
| Number of training programs over 30km from the residence area | None | 28 (45.2) |
|  | 1 | 18 (29) |
|  | 2 | 4 (6.5) |
|  | 3 | 3 (4.8) |
|  | > 3 | 6 (9.7) |
|  | Not attended any training program | 3 (4.8) |
| Nature of the training programs | All mandatory | 1 (1.6) |
|  | All non-mandatory | 26 (41.9) |
|  | Some are mandatory and others are non-mandatory | 32 (51.6) |
|  | Not attended any training program | 3 (4.8) |
| Training program subject | Life and Physical Sciences (Biology/Geology) | 54 (87.1) |
|  | Educational Sciences | 27 (43.5) |
|  | Teaching practice and Didactics | 25 (40.3) |
|  | Personal Education and Ethics | 8 (12.9) |
|  | Specific trainings | 15 (24.2) |
|  | Not attended any training program | 3 (4.8) |
| Attended Health Education training programs | Yes | 21 (33.9) |
|  | No | 41 (66.1) |
|  | Not attended any training program | 3 (4.8) |
| Institutions that promote training for teachers | Universities and associated labs | 30 (48.4) |
|  | Public institutions related to the Ministry of Education | 49 (79) |
|  | Other public institutions not related to the Ministry of Education | 5 (8.1) |
|  | Other institutions (Non-public) | 24 (38.7) |
|  | Not attended any training program | 3 (4.8) |

The items “Training program subject” and “Institutions that promote training for teachers” derived from a multiple-choice question. Teachers were allowed to select more than one option.

As we can see above, 77.4% (48) of teachers had attended three or more training programs and 64.6% (40) of them granted 3 or more credits. The most frequent, 32.3% (20) amount spent varies between € 100 and € 200 while 29% selected only free training program (18). Geographic location of the programs does not seem a determining factor for participation in training activities since half of the teachers attended programs more than 30km of their residential area. Also, most teachers, 51.6% (32) participated either in training programs that are mandatory and non-mandatory for their career. 87.1% (54) Life and Physical Sciences (Biology and Geology) is the area where trainees participated in more training programs with 87.1% (54), followed by the training programs in Educational Sciences, with 43.5% (27). Part of the trainees, 33.9% (21) also refers that have participated in specific training activities about Health Education especially on sexual education and sexually transmitted infections. These training programs are essential to keep teachers up to date about technical subjects. Institutions that promote the training are also an important issue with 79% (49) of the teachers choosing the institutions related to the Ministry of Education and Science and 48.4% (30) also select universities or associated labs to attend this kind of programs.
